# Ether-compatible sulfurized polyacrylonitrile cathode with excellent performance enabled by fast kinetics via selenium doping

**DOI:** 10.1038/s41467-019-08818-6

**Published:** 2019-03-04

**Authors:** Xin Chen, Linfeng Peng, Lihui Wang, Jiaqiang Yang, Zhangxiang Hao, Jingwei Xiang, Kai Yuan, Yunhui Huang, Bin Shan, Lixia Yuan, Jia Xie

**Affiliations:** 1 State Key Laboratory of Advanced Electromagnetic Engineering and Technology, School of Electrical and Electronic Engineering, Huazhong University of Science and Technology, 430074 Wuhan, China; 2 State Key Laboratory of Materials Processing and Die & Mould Technology, School of Materials Science and Engineering, Huazhong University of Science and Technology, 430074 Wuhan, China

## Abstract

Sulfurized polyacrylonitrile is suggested to contain S_n_ (*n* ≤ 4) and shows good electrochemical performance in carbonate electrolytes for lithium sulfur batteries. However inferior results in ether electrolytes suggest that high solubility of Li_2_S_n_ (*n* ≤ 4) trumps the limited redox conversion, leading to dissolution and shuttling. Here, we introduce a small amount of selenium in sulfurized polyacrylonitrile to accelerate the redox conversion, delivering excellent performance in both carbonate and ether electrolytes, including high reversible capacity (1300 mA h g^−1^ at 0.2 A g^−1^), 84% active material utilization and high rate (capacity up to 900 mA h g^−1^ at 10 A g^−1^). These cathodes can undergo 800 cycles with nearly 100% Coulombic efficiency and ultralow 0.029% capacity decay per cycle. Polysulfide dissolution is successfully suppressed by enhanced reaction kinetics. This work demonstrates an ether compatible sulfur cathode involving intermediate Li_2_S_n_ (*n* ≤ 4), attractive rate and cycling performance, and a promising solution towards applicable lithium-sulfur batteries.

## Abstract (web summary)

Lithium sulfur batteries are promising for advanced energy storage, but polysulfide shuttling limits performance lifetime. Here the authors report selenium-doping in a sulfur-based cathode to prevent dissolution of polysulfide intermediates, leading to ether compatibility, high capacity and stable cycling.

## Introduction

The increasing demand for advanced energy storage has attracted extensive research of lithium-ion batteries^1^. Lithium–sulfur(Li–S) batteries gain interest due to the high theoretical energy density of 2600 W h kg^−1^ ^2–4^^.^. However, the insulating nature of sulfur and end-product Li_2_S make them electrochemically inert, and the theoretical level is difficult to achieve^5–8^. Carbon sulfur composites with efficient conductive networks can usually deliver high capacity with good reactivity^9–13^. Nevertheless, it is challenging to maintain high capacity because of the dissolution of the lithium polysulfide intermediates Li_2_S_n_ followed by the “shuttling effect”, which lead to loss of active materials, low Coulombic efficiency and poor cycle life^14–17^. Interestingly, with different sulfur sources, a different set of Li_2_S_n_ (*n* = 2–8) will be generated in the redox reaction, in which the solubility and the redox conversion of Li_2_S_n_ play an important role in cathode performance^18–20^. For instance, when elemental sulfur is used, high order polysulfide intermediates (Li_2_S_n_ (*n* > 4)) with high solubility will be generated^21–23^. Since it is difficult to convert all soluble Li_2_S_n_ (*n* > 4) into insoluble Li_2_S_2_ or end-product Li_2_S without dissolution, it is very challenging to shut down the “shuttling effect” and obtain high Coulombic efficiency and satisfactory cycling performance^24,25^. On the other hand, if only intermediate (Li_2_S_2_), which is close to completely insoluble, is involved such as in the case of small sulfur molecule (S_2–4_) captured in microporous carbon, the dissolution of the polysulfides can be avoided due to a “quasi solid state” reaction mechanism^26^. Similar strategy has been employed with elemental sulfur cathodes in a recent work by Nazar and coauthors, which shows good capacity with low electrolyte/sulfur ratio and minimum dissolution of polysulfides^27^. However, it is reasonable to expect that without soluble polysulfide intermediates, the reaction kinetics as well as the rate capability will be limited presumably due to the intrinsic “quasi solid state” reaction mechanism^28–30^. Thus to develop sulfur cathodes with good capacity, long life and high rate, it is necessary to involve soluble polysulfide intermediates but mitigate polysulfides dissolution at the same time.

Surprisingly, sulfurized polyacrylonitrile (SPAN), reported by Wang and co-workers^31^, seems to be a promising cathode which exhibits good capacity, reasonable rate capability, nearly 100% Columbic efficiency, good cycling performance and presumably no polysulfides dissolution in carbonate electrolyte. Though the exact structure of SPAN is still not clear, it is generally believed that sulfur is chemically bonded to the pyrolyzed pyridine ring initially and during the chemical reaction, and Li2Sn (n ≤ 4) is involved in the redox reaction. Recently the performance of SPAN cathodes has been further improved by using modified versions, such as SPAN@MWCNT^32^, SPAN@GNS^33^, NiS_2_-SPAN^34^, and highly ordered MSPAN^35^ and carbonized SeS_2_-PAN^36^. Among them, SPAN prepared by Archer’s group exhibited high capacity and durability for 1000 cycles, but only in carbonate-based electrolytes^37^. It has been suggested that sulfur exists as –S_x_^2-^– (2 ≤ *n* ≤ 4) units in S@pPAN, and there are no soluble intermediates during the redox process of S@pPAN in carbonate-based electrolyte^38^. Nevertheless, SPAN cathodes usually work well in carbonate electrolyte but show poor results in ether electrolyte^37,39–41^. It is reasonable to speculate that in carbonate electrolyte, Li_2_S_n_ (*n* ≤ 4) shows lower solubility and can react with carbonate to form a protective solid electrolyte interphase (SEI) to mitigate the dissolution problem^42^. But in ether electrolyte the solubility of Li_2_S_n_ (*n* ≤ 4) is high enough to trump the limited redox conversion and leads to the dissolution and the shuttling effect^28,30^, which suggests that an underlying slow redox conversion and slow kinetics problem still exists despite that ether-based electrolytes evidently have better compatibility with lithium metal anodes^43,44^. Thus, it is desirable to accelerate the redox conversion of Li_2_S_n_ (*n* ≤ 4) and boost the kinetics in SPAN, which should mitigate polysulfides dissolution and lead to compatibility with both carbonate and ether electrolyte as well as high rate and long cycle life performance.

Similar with S, selenium (Se) lies in the same column with S in the periodic table and shows much better kinetics in high-rate Se-based or Se-doped composites^45–49^. Recent work by Qian et al. shows that a catalytic amount of Se doping in an elemental sulfur carbon composite leads to a tremendous rate-boosting effect^50^. Different from other rate accelerators such as conducting carbons^51^, metal oxides^3^, metal sulfides^23^, and metal nitrides^52^, Se can not only easily achieve uniform distribution at a molecular level through Se–S bonding, but also contribute capacity. However, such a concept has only been shown in elemental sulfur cathodes in which a large amount of conducting carbon is still used and the cathode capacity is limited.

Herein, we design Se_x_SPAN (*x* = 0.06, 0.09, 0.14) composites as the cathode with the intention of using a catalytic amount of Se as both a rate accelerator and a capacity contributor in a polymeric framework. By accelerating the redox transformation of the only low order intermediate Li_2_S_n_ (*n* ≤ 4) into insoluble Li_2_S_2_ or Li_2_S, the dissolution problem should be largely mitigated. Indeed the experiment results show that compared with traditional SPAN, Se_0.06_SPAN cathode delivers high reversible capacity, superior rate performance and long cycles in both ether and carbonate electrolytes. The high rate performance could be attributed to higher electronic conductivity and faster lithium ion diffusion by Se-doping, which successfully serves as both a capacity contributor and a rate promotor in sulfurized polyacrylonitrile cathodes. As a result, the dissolution of polysulfides is mitigated in sulfurized polyacrylonitrile cathodes. This work not only demonstrates an ether-compatible sulfurized polyacrylonitrile cathode enabled by fast kinetics, to the best of our knowledge, one of the best rate and cycling performance simultaneously achieved, but also shows a sulfur cathode involving soluble polysulfides without polysulfide dissolution and shuttling. Such an approach provides a promising solution towards practical lithium sulfur batteries.

## Results

### Synthesis and characterization

**Fig. 1:**
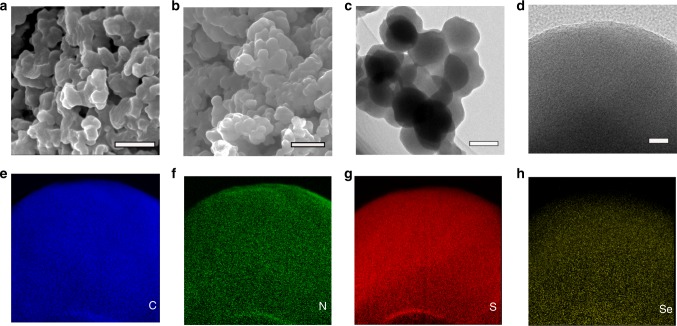
Materials characterization. SEM images of **a** pPAN. **b** Se_x_SPAN composites. **c** TEM image of Se_x_SPAN composite. **d** EDS elemental mapping images of the Se_x_SPAN composite, marked by a square, for carbon (**e**), nitrogen (**f**), sulfur (**g**), and selenium (**h**). Scale bars, 500 mm (**a**, **b**), 200 mm (**c**), 20 nm (**d**)

The scanning electron microscopy (SEM) images show the morphology of pyrolyzed PAN (pPAN) and Se_0.06_SPAN composites (Fig. 1a, b). The pPAN synthesized under Ar atmosphere at 300 °C is composed of irregular particles with a size around 500 nm. The Se_0.06_SPAN composite prepared by annealing the mixture of Se_x_S and PAN (3:1 by weight) at the same method is consisted of round particles with a size around 200 nm. The morphology difference is attributed to the dehydrogenation reaction between PAN and Se_x_S. Se_x_S can dehydrogenate PAN to generate a conductive framework by firstly forming stable heterocyclic rings which is further dehydrogenated and substituted by Se_x_S to form crosslinking C–S–Se_x_–S–C chains or ring structures. The transmission electron microscopy (TEM) images of Se_0.06_SPAN composites (Fig. 1c) show that original particles are in circular-shape with a size around 200 nm, and these particles aggregate into a bulky cluster, which is consistent with the SEM images. High resolution transmission electron microscopy (HRTEM) is used to explore the microstructure of the Se_0.06_SPAN composite. It can be clearly observed that Se_0.06_SPAN composite has a uniform structure. The energy-dispersive X-ray spectroscopy (EDS) elemental mapping images reveal that the carbon elemental mapping image overlaps with sulfur and selenium mapping images, suggesting the homogeneous distribution in the Se_0.06_SPAN composite (Fig. 1d–h). The content of Se and S in the Se_0.06_SPAN composite is 47.25% as shown in Supplementary Table 1, containing Se_0.09_SPAN and Se_0.14_SPAN composites with different doped proportions of Se. In the thermogravimetric analysis (TGA), the Se_x_SPAN composites show almost no weight loss below 400 °C (Supplementary Fig. 1).

**Fig. 2:**
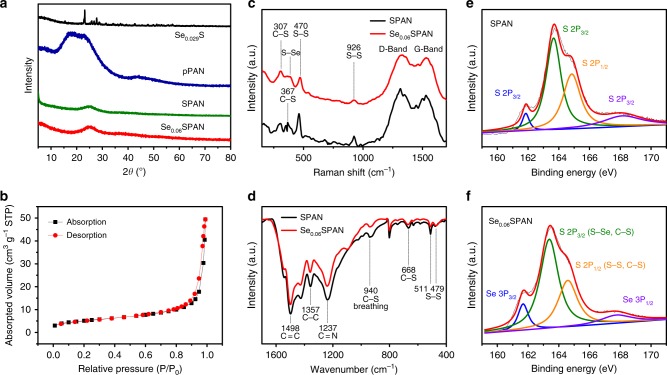
Materials characterization. **a** XRD patterns of Se_0.029_S, pPAN, SPAN, and Se_0.06_SPAN. **b** N_2_ adsorption–desorption isotherms of Se_0.06_SPAN. **c** Raman spectra **d** FTIR spectra of SPAN and Se_0.06_SPAN. XPS analyses (S2p) of SPAN (**e**) and Se_0.06_SPAN (**f**) composites, respectively

The phase structure of Se_0.06_SPAN composites is explored by X-ray diffraction, Raman spectroscopy and FT-IR spectra. The XRD patterns of pPAN show one broad peak range from 17 to 26 degree (Fig. 2a). While pPAN shows a major peak at 2*θ* = 17° corresponding to the (110) plane of the PAN crystalline structure, in the case of SPAN, such characteristic peak of pPAN disappears and a broad diffraction peak at 2*θ* = 25° corresponding to the graphitic (002) plane newly presents after pyrolysis with sulfur^53^. The XRD patterns show quite different results between Se_0.029_S and Se_0.06_SPAN. The latter shows an amorphous structure. The XRD peaks of Se_0.029_S are very similar with that of Se_0.06_S (Supplementary Fig. 6). Moreover, the XRD peaks of Se_0.06_S can not be observed in the XRD peaks of Se_0.06_SPAN. It suggests that there is no Se_0.06_S in Se_0.06_SPAN composite. Although the proportions of Se are different, the Se_0.09_SPAN and Se_0.14_SPAN composites show similar patterns (Supplementary Fig. 3). The XRD patterns of Se_0.06_SPAN composite show one broad peak at 25 degree and no Se_0.029_S peak is detected, which suggests that the formation of an amorphous structure in Se_0.06_SPAN composite materials. It is noteworthy that the proportion of Se has been increased obviously from 0.029 to 0.06, which suggests that the dehydrogenation reaction is mainly caused by sulfur. The sorption analysis using Brunauer–Emmett–Teller (BET) theory (Fig. 2b) reveals a relatively low surface area of 18 m^3^ g^−1^.

To analyze the chemical bonds of the Se_0.06_SPAN composite, Raman spectra, Fourier-transform infrared spectroscopy (FT-IR) and X-ray photoelectron spectroscopy (XPS) are performed (Fig. 2c–f, Supplementary Fig. 4, 5). Specific peak assignments are summarized (Supplementary Fig. 4, 5 and Supplementary Tables 4, 5). As the previous reports^37^, the peaks of SPAN located at 307 and 367 cm^−1^ correspond to the C−S bonds and those at 470 and 926 cm^−1^ correspond to the S−S stretch. However, the peak at 387 cm^−1^ corresponding to the S−Se bond^50^ appears in Se_0.06_SPAN, instead of C−S bond. From the results of Raman spectra, the structure of Se_x_SPAN is very similar with that of SPAN. Moreover, the Raman spectra provide information on the degree of crystallization of materials. It has been known that representative Raman spectra of carbon-based materials show two major peaks at 1325 and 1532 cm^−1^, corresponding to the disordered D band and the graphitic G band, respectively. According to the intensity ratio (*I*_G_/*I*_D_) of these two bands, Se_0.06_SPAN shows a higher degree graphitization than that of SPAN, as the *I*_G_/*I*_D_ ratios for Se_0.06_SPAN and SPAN are 0.98 and 0.89, respectively. Meanwhile, the electronic conductivity of Se_x_SPAN more than doubled compared to SPAN through direct current (DC) polarization method (Supplementary Fig. 2 and Supplementary Table 3). On the basis of the information, the structure of the Se_0.06_SPAN can be confirmed to be a carbon construction via dehydrogenation and efficient π–π stacking, with sulfur covalently bonded to the carbon backbones simultaneously, which is also consistent with the XRD analysis.

Figure 2d shows the FT-IR spectra of SPAN and Se_0.06_SPAN. It is noteworthy that the peaks are almost same between the two spectra. The peaks at 479 and 511 cm^−1^ correspond to the S−S stretching, and the peak at 668 cm^−1^ corresponds to the C−S stretching. Besides, the peak at 940 cm^−1^ corresponds to the ring breathing in which a C−S bond is included. Therefore, these characteristic peaks verify the structure of C−S and S−S bonds after the dehydrogenation reaction. Moreover, the FT-IR analysis give information on the atom distribution of the carbon backbones. It has been known that the peaks at 1237 and 1498 cm^−1^ and of Se_0.06_SPAN correspond to the symmetrical stretching of C = N and C = C bonds, respectively^37,53^, and the peak at 801 cm^−1^ corresponds to the ring breathing of six-membered rings^37,53^. Apparently, the structure of Se_x_SPAN composite is very similar with that of SPAN from the FT-IR analysis, which is consistent with the Raman analysis. These suggest that Se_x_SPAN also has complicated structure as well as SPAN.

As shown in Fig. 2e, there are two S2p peaks located at 163.6 and 164.9 eV, corresponding to the S−S and C–S bonds, respectively^34^. In addition, S2p peaks are also apparent at 161.8 and 168.1 eV. As shown in Fig. 2f, there are two S2p peaks located at 163.3 and 164.6 eV, corresponding to the S−Se and S−S homopolar bonds, respectively^54,55^. The two S2p peaks also attribute to the C−S bond. Owing to the lower electron density of sulfur, the two binding energies at 163.4 and 164.6 eV represent a −0.2 and −0.3 eV shift for the S–Se and C–S bonds, respectively^54,55^. The two binding energies at 161.6 and 167.7 eV also represent a −0.2 and −0.4 eV shift, which also validat the interaction between Se and S in the Se_0.06_SPAN^54,55^. According to the XPS spectrum (Supplementary Fig. 7a), the predominant peaks of C1s, N1s, S2p, and Se3d all exist. Meanwhile, the presence of Se−S bond is also confirmed by the Se3d XPS spectra. A doublet Se3d peak with binding energy of 55.6 and 56.4 eV is attributed to the S−Se bond, and this peak is curve-fitted (Supplementary Fig. 7b).

### Electrochemical performance of cathodes

**Fig. 3:**
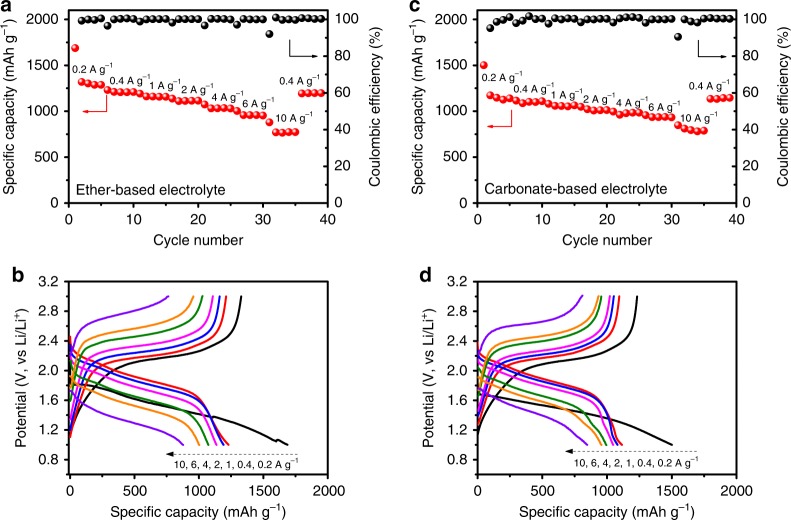
Electrochemical performance of the lithium–sulfur batteries. **a** Rate performance of the Se_0.06_SPAN measured at various current densities in ether-based electrolyte. The current densities are same for both charge and discharge in each cycle. **b** The corresponding electrochemical discharge and charge profiles of Se_0.06_SPAN at various cycles. **c** Rate performance of the Se_0.06_SPAN measured at various current densities in carbonate-based electrolyte. **d** The corresponding electrochemical discharge and charge profiles of Se_0.06_SPAN at various cycles

The rate performance of Se_0.06_SPAN composite cathodes is investigated in both ether-based and carbonate-based electrolytes (Fig. 3), with 1 mg cm^−2^ active material loading. When cycling at a current density of 0.2 A g^−1^ (0.13C) in ether-based electrolyte, the Se_0.06_SPAN composite cathodes deliver an initial capacity of 1680 mA h g^−1^ based on S and Se. The Se_0.06_SPAN composite cathodes show significantly excellent rate performance in both ether and carbonate-based electrolytes. In ether-based electrolyte, they deliver a reversible capacity of 1320, 1210, 1160, 1110, 1030, 960, and 900 mA h g^−1^ with the increasing current rate from 0.2 (0.13C), 0.4 (0.26C), 1 (0.65C), 2, 4, 6 to 10 A g^−1^ (6.5C) respectively (Fig. 3a, b), corresponding to an excellent utilization ratio (84%) to its theoretical capacity (1546 mA h g^−1^, Supplementary Table 2). The capacity then increases back to 1228 mA h g^−1^ when the current density returns back to 0.4 A g^−1^, demonstrating its superior stability to bear current changes. The reversible discharge capacities are slightly lower in the carbonate-based electrolyte, varying from 1173, 1115, 1081, 1048, 995, 957 to 847 mA h g^−1^ with the increasing current rate from 0.2, 0.4, 1, 2, 4, 6 to 10 A g^−1^ respectively (Fig. 3c, d). When the current density returns to 0.4 A g^−1^ after cycling at various rates, the discharge capacity of Se_0.06_SPAN composite cathode can recover to 1135 mA h g^−1^. In contrast, the discharge capacities of traditional SPAN cathode decreases significantly at a varied current density in ether-based electrolyte (Supplementary Fig. 8b). These results indicate that the Se doping played a critical role for enhancing the rate performance by accelerating the reaction kinetics. It is clearly shown that the rate performance of the Se_0.06_SPAN composite cathode is higher than that of literature reports (Supplementary Fig. 13a and Supplementary Table 6).

**Fig. 4:**
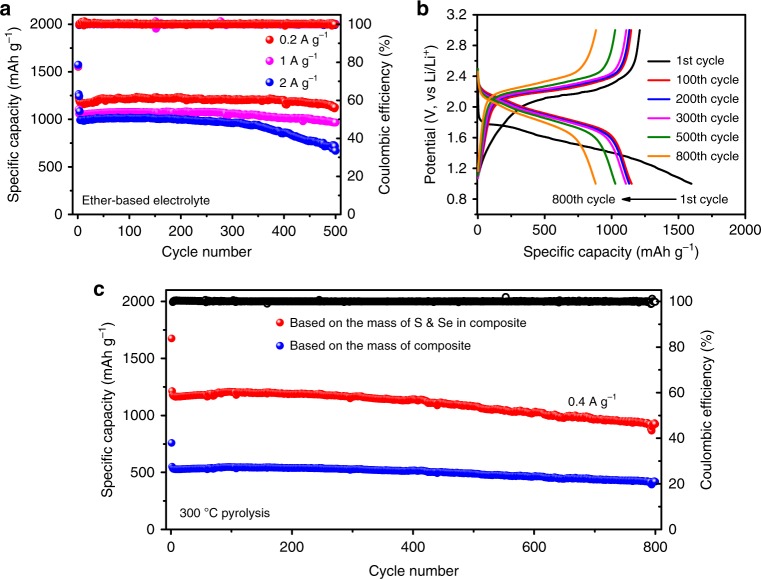
Electrochemical performance of the lithium–sulfur batteries. **a** Cycle performance of Se_0.06_SPAN at various current densities in ether-based electrolyte. **b** Electrochemical discharge and charge profiles of Se_0.06_SPAN at various cycles. The tests are performed at 0.4 A g^−1^. **c** Capacity and Coulombic efficiencies versus cycle number for Se_0.06_SPAN. The red curve report capacities relative to the weight of the S and Se in the cathode, whereas the data represented by blue curve are the corresponding capacities based on the overall composite mass

The cycling performance of the cells with Se_0.06_SPAN composites cathode in ether-based electrolyte at a current density of 0.2 A g^−1^ (0.13C), 1 A g^−1^ (0.65C), 2 A g^−1^ (1.3C) is shown in Fig. 4a. The reversible capacities of the cells are 1230, 1100, 1200 mA h g^−1^ in the second cycle respectively. After 500 cycles, the cells with Se_0.06_SPAN composites cathode maintain a reversible capacity of 1130 mA h g^−1^ at 0.2 A g^−1^, possessing the capacity retention of 91.6% with almost 100% Coulombic efficiency based on the second discharge capacity. Apparently, it shows good compatibility with ether-based electrolytes. In contrast, the discharge capacity of traditional SPAN cathode decays significantly in ether-based electrolyte (Supplementary Fig. 8a). The phenomenon for traditional SPAN cathode in ether-based electrolyte can also be seen in other literature reports^37,42^. The Se_0.06_SPAN cathode is firstly cycled for 10 cycles with LiNO_3_, and then cycled in ether-based electrolyte without LiNO_3_. The cycle performance of Se_0.06_SPAN cathode is still good even without LiNO_3_ (Supplementary Fig. 8c, d). It suggests that the superior electrochemical performance of Se_0.06_SPAN cathode is only caused by the introduction of catalytic Se. Thus, the LiNO_3_ additive in ether-based electrolyte only has an effect on the protection of Li metal anode to achieve better electrochemical performance. The high reversible capacity, long cycle life and high Coulombic efficiency of Se_0.06_SPAN composites cathodes demonstrate that the Se can accelerate redox kinetics and prevent the dissolution of the lithium polysulfides effectively. The reversible capacities of Se_0.06_SPAN composites cathode with 1 mg cm^−2^ active material loading are 1150, 1131, 1107, 1028, and 881 mA h g^−1^ based on S and Se after 100, 200, 300, 500, and 800 cycles with a current density of 0.4 A g^−1^ (0.26C), respectively (Fig. 4b). The reversible capacity of the cell is 1156 mA h g^−1^ based on S and Se in the second cycle (Fig. 4c). The capacity degradation upon repeated cycles of discharge and charge is only 0.029% per cycle from the second to 800th cycle, which hit a record among all reports cycled in ether-based electrolyte. Even up to 3 mg cm^−2^ based on the mass of S and Se, the Se_0.06_SPAN composite cathodes still deliver a high specific capacity (Supplementary Fig. 9d). The Se_0.09_SPAN and Se_0.14_SPAN composite cathodes also show good cycle performance (Supplementary Fig. 9a, b) but slightly lower capacity due to higher proportion of Se (Supplementary Fig. 9c).

It is clearly shown that the cycle performance of Se_0.06_SPAN composite cathode is higher than the representative values of literature reports (Supplementary Fig. 13b and Supplementary Table 7). Based on the mass of the whole mass of Se_0.06_SPAN, the cathode still delivers a capacity of 546 mA h g^−1^ at second cycle, corresponding to an excellent utilization ratio (75%) to its theoretical capacity (726 mA h g^−1^), and 416 mA h g^−1^ even after 800 cycles based on the Se_0.06_SPAN composite (Fig. 4c). Compared to literature reports, the electrochemical performance of Se_0.06_SPAN composite cathode is at the top level. These results clearly demonstrate that the application of Se in Se_0.06_SPAN composite not only accelerate redox kinetics but also improve the cycle stability of the cells. In addition, the Coulombic efficiencies of the cells with Se_0.06_SPAN cathodes all remain at nearly 100% (except for the first cycle).

The cathode with 2.3 mg cm^−2^ active material loading cycling in ether-based electrolyte is shown in Supplementary Fig. 10a. After 50 cycles, the cycled CR2025 coin cells in two different electrolytes are unpacked. The separator for the electrode cycled in ether-based electrolyte shows less color (Supplementary Fig. 11). It may be that the Se_0.06_SPAN composites hardly dissolve in ether-based electrolyte. Furthermore, the surface of Li metal from the cell cycling in carbonate-based electrolyte is darkened. The SEM image of Li metal is much smoother in ether-based electrolyte (Supplementary Fig. 12) than in carbonate-based electrolyte, which is an indication for better compatibility of ether-based electrolyte with Li metal.

## Discussion

**Fig. 5:**
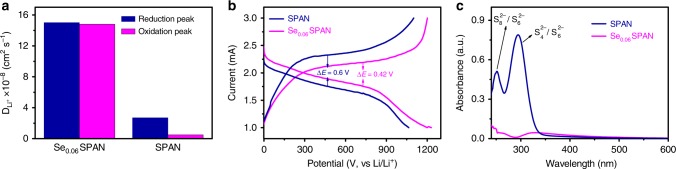
Improved electrochemical reaction kinetics. **a** Li^+^ diffusion coefficients of reduction peak of Se_0.06_SPAN and SPAN cathodes. **b** Discharge–charge curves of SPAN and Se_0.06_SPAN electrodes. **c** UV–Vis absorption spectra of the solution after washing cycled SPAN and Se_0.06_SPAN cathodes

The lithium ion transference is another factor to affect battery performance. The lithium ion diffusion coefficients (*D*_Li_^+^) for Se_0.06_SPAN are quantitatively calculated by a series of cyclic voltammetry (CV) with different scan rates. Randles–Sevick equation^56^ is adopted, and then lithium ion diffusion coefficient is calculated based on the slop of the linear plot of the peak current (*I*_p_) versus the square root of the scan rate (*v* ^0.5^). From the linear relationship of *I*_p_ and *v*^0.5^ (Supplementary Fig. 14), *D*_Li+_ of two reduction peaks are obtained (Fig. 5a). It is worth noting that *D*_Li+_ for the reduction and oxidation peaks of the Li–Se_0.06_SPAN battery are both higher than the Li–SPAN battery, suggesting that Se-doping facilitates fast Li-ion transport. This finding can be contributed to the catalytic effect of Se on the electrochemical performance for lithium storage, which is consistent of the superior rate performance and cycle performance of Li–Se_x_SPAN batteries. A comparison of reduction potentials is also studied to further analyze the effect of Se (Supplementary Fig. 15a). The reduction peaks for Se_x_SPAN electrode are 2.07 and 1.77 V, lower than that of pure sulfur electrode (2.32 and 2.02 V), but higher than that of S/microC electrode (1.68 V). The two peaks at 2.32 and 2.02 V correspond to the reduction of S_8_ to higher order polysulfides (Li_2_S_n_, 4 < *n* < 8) and then to lower order polysulfides (Li_2_S_n_, *n* ≤ 4)^30,57^. The 1.68 V peak is related to the reduction of smaller sulfur molecules confined within the microporous carbon (from S_2_ to Li_2_S_2_/Li_2_S)^28,30^. Because of the unique sulfur structure (–S_x_–, 2 ≤ *x* ≤ 4) in SPAN^38^, the 2.07 and 1.77 V peaks correspond to the fast reduction of soluble Li_2_S_n_ (*n* ≤ 4) and then to insoluble Li_2_S_2_/Li_2_S. The typical reduction peaks of Se_x_SPAN, pure sulfur, and S/microC are all in agreement with their own galvanostatic discharge curves (Supplementary Fig. 15b). The 1.8 V average plateau is observed for Se_x_SPAN, which also falls in between elemental sulfur and small molecule sulfur cathodes. It also suggests that only soluble and lower order of polysulfides (Li_2_S_n_, *n* ≤ 4) are involved. Thus the CV curves and voltage profiles suggest a reaction mechanism involving transition between soluble Li_2_S_n_ (*n* ≤ 4) and insoluble Li_2_S_2_/Li_2_S during the lithiation and delithiation process. Owing to the fast Li-ion diffusion and reaction kinetics enabled by Se-doping, the soluble polysulfides can be transformed effectively, which inhibit polysulfides dissolution and the shuttling effect. Figure 5b shows discharge/charge voltage profiles of SPAN and Se_0.06_SPAN electrode at 0.2 A g^−1^. The Se_0.06_SPAN electrode has a discharge capacity of 1240 mA h g^−1^, much higher than that of SPAN electrode. Moreover, the Se_0.06_SPAN electrode possesses a relatively low polarization value of 0.42 V between the charge and discharge plateaus, which is much lower than that of 0.6 V for the SPAN. The improved discharge capacity and reductive polarization show that Se_0.06_SPAN is able to boost the electrochemical reaction kinetics during the discharge/charge processes in Li–S batteries.

To validate the above-mentioned points, we investigate the Li^+^ migration within SPAN and Se_x_SPAN using density functional theory (DFT) model. The experimental data show that the migration energy barriers within SPAN and Se_x_SPAN are 1.33 and 0.39 eV, respectively (Supplementary Fig. 16). It is apparently that the structure of Se_x_SPAN is more beneficial for Li migration. The lower migration energy barrier is in agreement with the experimental results that the D_Li_^+^ of Se_x_SPAN is higher than that of SPAN. This finding likely explains that Se_x_SPAN has better reaction kinetics compared with SPAN. A lower barrier can lead to an increase in the diffusion rate according to the exponential rule. Faster Li^+^ migration within the Se_x_SPAN can promote the redox reaction of soluble intermediates (Li_2_S_n_, *n* ≤ 4), thus effectively to mitigate polysulfides dissolution and the shuttling effect.

**Fig. 6:**
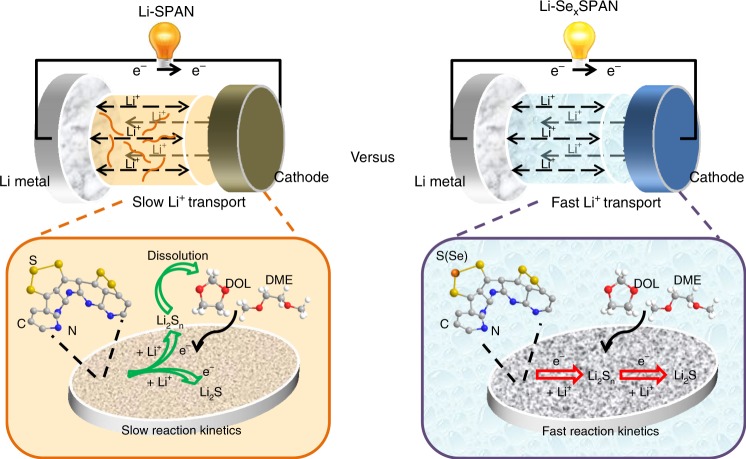
The scheme of proposed reaction process. Small amount of Se-doping significantly enhances the redox conversion of polysulfides and reaction kinetics, leading to ether-compatibility and superior performance of Li–Se_0.06_SPAN battery

The dissolution and diffusion of polysulfides during the redox process of SPAN and Se_0.06_SPAN cathodes are also investigated in ether-based electrolyte. The Li–SPAN and Li–Se_0.06_SPAN batteries after several cycles are both discharged to 2.0 V. Then, the SPAN and Se_0.06_SPAN cathodes are both rinsed with ether-based electrolyte after the batteries are unpacked. The solutions are measured by UV–Vis spectroscopy. As shown in Fig. 5c, for SPAN, the dissolution of polysulfides can be clearly detected in ether-based electrolyte in despite of undetectable polysulfides in carbonate-based electrolyte^37^. The SPAN for UV–Vis spectra test is prepared at 450 °C for 6 h by the same synthetic method according to the previous literature^37^. The sharp peak at ~280 nm corresponds to the S_4_^2−^/S_6_^2−^ species, the shoulder peak located at ~350 nm corresponds to the S_4_^2−^ species, and the peak at 250 nm to the S_8_^2−^/S_6_^2−^ species, in which S_x_^2−^ (*x* = 4–8) species are presumably generated from low order intermediates S_n_^2−^(*n* ≤ 4)^58,59^. Above results suggest that in ether-based electrolyte the dissolution and diffusion of polysulfides from the SPAN cathode occur, resulting in rapid capacity decay. However, polysulfides are undetectable for Se_0.06_SPAN cathode, suggesting that fast redox conversion of polysulfide intermediates and fast kinetics of the Se_0.06_SPAN cathode in ether-based electrolyte. It is reasonable to conclude that the catalytic amount of Se-doping significantly enhances the redox conversion of polysulfides and reaction kinetics, thus leading to the compatibility with ether-based electrolyte and the outstanding electrochemical performance of Li-Se_0.06_SPAN batteries (Fig. 6). Similar rate boosting effects are seen in both Se and Tellurium (Te), which indicates the mechanism may be related to the promotion of electron transfer by Se and Te since Te is a well-known catalyst to promote electron transfer in organic chemistry^60^. Further studies of detailed mechanism and performance optimization are still ongoing.

In summary, we design a selenium-doped sulfurized polyacrylonitrile (Se_x_SPAN, *x* < 0.15, ~50 wt% Se_x_S) cathode which shows excellent electrochemical performance and outstanding compatibility with ether-based electrolyte. The catalytic amount of Se-doping leads to higher lithium ion diffusion coefficient, relatively low polarization, resulting in rapid conversion of polysulfide intermediates and fast reaction kinetics, which in turn prevent the dissolution of polysulfide intermediates in ether. It is believed that the solution by rapid and complete conversion from soluble Li_2_S_n_ (*n* ≤ 4) to insoluble Li_2_S can also be applied in other similar cathodes involving Li_2_S_n_ (*n* ≤ 4). The solution should also be universal for all sulfur cathodes involving Li_2_S_n_ (*n* ≤ 4). This work shows a sulfur cathode involving intermediate Li_2_S_n_ (*n* ≤ 4) with excellent compatibility with ether-based electrolyte, simultaneously achieved rate and cycling performances that are among the best for sulfur cathodes, to the best of our knowledge, and a promising solution towards applicable lithium sulfur batteries.

## Methods

### Synthesis of selenium-sulfur composites

Commercial sulfur and selenium powders are ball-milled at a weight ratio of 15:1, 12:1, and 10:1 with ethanol as the dispersant. The resulting mixture is dried at 60 °C for 6 h in a vacuum oven. Then the mixture is fully filled in a 200 mL Teflon-lined stainless steel autoclave, heated at 250 °C for 12 h and then cooled to room temperature naturally. The mole and weight ratio of sulfur and selenium in Se_x_S will reduce slightly during the preparation compared to the initial mixing ratio.

### Synthesis of selenium-doped sulfurized polyacrylonitrile

A Se_x_S and PAN mixture is milled uniformly at a weight ratio of 3:1 for 0.5 h in a mortar and then annealed at 300 °C for 2.5 h in argon atmosphere. During this step, slight excess amount of Se_x_S is used. A part of Se and S will be removed from the reactant either in S or Se gas form or H_2_S gas form generated in dehydrogenation reaction at 300 °C. Since S is easier to evaporate than Se, the resulting S:Se ratio will be lower than the initial mixing ratio. For the PAN/S composites without selenium, the preparation procedure is similar, except the adding selenium.

### Material characterization

X-ray powder diffractometer (PANalytical X'pert PRO-DY2198, Holland) is used to collect X-ray diffraction (XRD) patterns, operating at 40 kV and 40 mA using Cu Ka radiation (*λ* = 0.15406 nm) and determining structure refinement of the XRD patterns using GSAS software. The test of Brunauer–Emmett–Teller (BET) surface area is measured at −196 °C by a BET analyzer (Micromeritics ASAP 2010, US). Scanning electron microscopy (SEM) (FEI, Sirion 200) is used to collect images of pPAN and Se_x_SPAN samples. Transmission electron microscopy (TEM) (FEI, Tecnai G200) is used to collect images of Se_x_SPAN samples. Raman spectra are measured by LabRAM HR800 (Horiba Jobin Yvon). Fourier Transform Infrared Spectra are taken using a Bruker Vertex 70 FTIR spectrometer. The X-ray photoelectron spectroscopy (XPS) analysis of Se_x_SPAN and SPAN materials is conducted with a Kratos Analytical spectrometer (AXIS-ULTRA DLD-600W) and an Al Kα (1486.6 eV) X-ray source, and the binding energy values are calibrated using the C 1s peak at 285.0 eV. The thermal gravimetric (TG) profiles are determined by thermal gravimetric analysis (PerkinElmer) in an argon atmosphere with a heat rate of 10 °C min^−1^ from 25 to 1000 °C. The UV–Vis spectrum are characterized by UV-2550 spectrophotometer (SHIMADZU). To reveal the electronic conductivities of SPAN and Se_x_SPAN, symmetric cells with configurations of Au/SPAN/Au and Au/Se_x_SPAN/Au are tested by Solartron 1470E CellTest system.

### Electrochemical measurements

All of the electrochemical measurements are tested on 2032 coin cells with lithium foil as the anode which assemble under an argon-filled glove box (H_2_O, O_2_ < 1 ppm). A working electrode is prepared by mixing Se_x_SPAN composites, super P, sodium carboxyl methyl cellulose (NaCMC) and styrene butadiene rubber (SBR) at a weight ratio of 80:10:5:5. The slurry is coated onto Al foil and then dried in a vacuum oven at 70 °C for 12 h. The active material density of each cell is determined to be 1–3 mg cm^−2^. A metallic Li sheet is used as the counter electrode, and 1 M LiPF_6_ in a mixture of ethylene carbonate/dimethylcarbonate (EC/DMC; 1:1 by volume) as the electrolyte (Zhuhai Smooth way Electronic Materials Co., Ltd (China)). The amount of electrolyte is about 60 μL in each cell during assembling. The ether electrolyte is 1 mol L^−1^ LiTFSI in a mixed solution of 1,2-dimethoxyethane (DME) and 1,3-dioxolane (DOL) (1:1 v/v) with LiNO_3_ (2 wt%) as the additive. Electrolyte is added to each coin cell at the amount of 30 μL in each cell during assembling. The electrochemical workstation (CHI614b) is used to test cyclic voltammetry (CV)profiles at a scan rate of 0.05 mV s^−1^ from 1 to 3 V at room temperature. The battery measurement system (Land, China) is used to measure the electrochemical performance and profiles from 1 to 3 V.

### Lithium ion diffusion coefficient

Lithium ion diffusion coefficients for Se_x_SPAN are calculated by a series of cyclic voltammograms at different scan rates, and the peak current data are analyzed with Randles-Sevick equation as following: *I*_p_ = 2.69 × 10^5^ *n*^1.5^ *AD*_Li_^+ 0.5^ *C*_Li_ *v*^0.5^ in which *D*_Li_^+^ represented lithium ion diffusion coefficient (cm^2^ s^−1^), *I*_p_ stood for the peak current in ampere (*A*), *n* is the number of electrons involved in the reaction (*n* = 2 for Li–S battery), *A* is the geometric area of the active electrode (cm^2^), *C*_Li_ referred to the lithium ion concentration (mol L^−1^) and *v* represented the scanning rate (V s^−1^).

### DFT calculations

The migration energy barriers reported herein were calculated using DFT within Perdew-Burke-Ernzerhof (PBE) generalized gradient approximation (GGA) via Vienna Ab initio Simulation Package (VASP). Projector augmented wave (PAW) method is used to depict the electron-ion interactions. The cutoff energy of 500 eV and Monkhorst−Pack k-meshes of 3 × 1 × 1 is set for the calculations. Vacuum layers of at least 10 Å is needed for non-periodic directions. The Gaussian broadening with a width of 0.05 eV is used for the integration of the first Brillouin zone. The structure is optimized until the Hellmann-Feynman force is smaller than 0.05 eV Å^−1^. Climbing-image nudged elastic band (CI-NEB) method is used to find the Li migration energy barriers.

## Supplementary information

Supplementary Information

Peer Review File

## Data Availability

The data that support the findings of this study are available from the corresponding authors upon request.
