# Supplementary material for: Ether-compatible sulfurized polyacrylonitrile cathode with excellent performance enabled by fast kinetics via selenium doping
Source: Nat Commun. 2019 Mar 4;10:1021. doi: 10.1038/s41467-019-08818-6 (PMC6399341; doi:10.1038/s41467-019-08818-6)
Supplement: Supplementary file 1 — Supplementary Information [file 41467_2019_8818_MOESM1_ESM.pdf]

Ether-compatible Sulfurized Polyacrylonitrile Cathode with Excellent Performance Enabled by Fast Kinetics *via* selenium-doping

Chen, et al[1,2], Linfeng Peng[1], Lihui Wang [1], Jiaqiang Yang[2], Zhangxiang Hao[2], Jingwei Xiang [2], Kai Yuan [2], Yunhui Huang[2], Bin Shan[2], Lixia Yuan*[, 2] & Jia Xie*[,1]

Affiliations:

[1] State Key Laboratory of Advanced Electromagnetic Engineering and Technology, School of Electrical and Electronic Engineering, Huazhong University of Science and Technology, Wuhan 430074, China

[2] State Key Laboratory of Materials Processing and Die & Mould Technology, School of Materials Science and Engineering, Huazhong University of Science and Technology, Wuhan 430074, China

Correspondence and requests for materials should be addressed to J. Xie (email: xiejia@hust.edu.cn) or to L. Yuan (email: yuanlixia@hust.edu.cn).

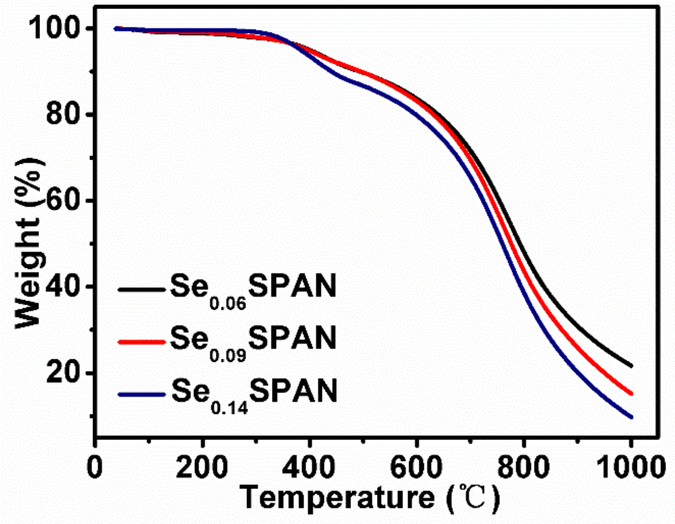

Supplementary Figure 1. **Thermogravimetric analysis of Se_xSPAN.** All tests were conducted from 25 to 1000 °C with a rate of 10 °C min^{-1} in N_2 atmosphere.

Supplementary Table 1. The C, N, S and Se content in the composite based on the elemental analysis.

Materials	C (%)	N (%)	S (%)	Se (%)	S&Se (%)	S/Se
$Se_{0.06}SPAN$	38.5	14.25	40.935	6.315	47.25	1/0.06
$Se_{0.09}SPAN$	38.01	14.14	38.798	9.052	47.85	1/0.09
$Se_{0.14}SPAN$	37.3	13.93	35.894	12.876	48.77	1/0.14

Supplementary Table 2. Theoretical capacity of Se_xSPAN composite.

Materials	Se (wt%) /S (wt%)	S&Se (wt%) in composite	Theoretical capacity (mAh g^{-1}, based on Se and S)	Theoretical capacity (mAh g^{-1}, based on the composite)
$Se_{0.06}SPAN$	13/87	47.25	1546	726
$Se_{0.09}SPAN$	19/81	47.85	1485	710
$Se_{0.14}SPAN$	26/74	48.77	1415	690

Supplementary Table 3. Electric conductivity results of SPAN and $Se_{0.06}SPAN$ using direct current (DC) polarization method.

Material	Resistance	Length (cm)	Area (cm^{-2})	Electric conductivity (S/cm)
SPAN	4.7×10^{7}	0.089	0.785	2.4×10^{-9}
$Se_{0.06}SPAN$	1.8×10^{7}	0.083	0.785	5.8×10^{-9}

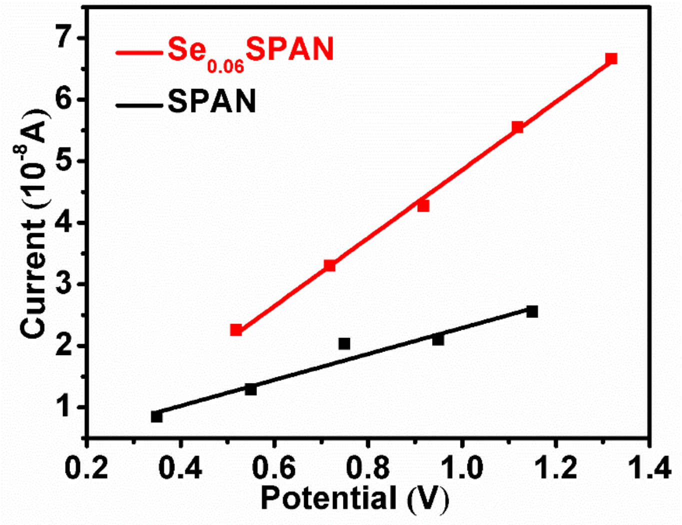

Supplementary Figure 2. Equilibrium current of the $Se_{0.06}$SPAN and SPAN at different set voltages.

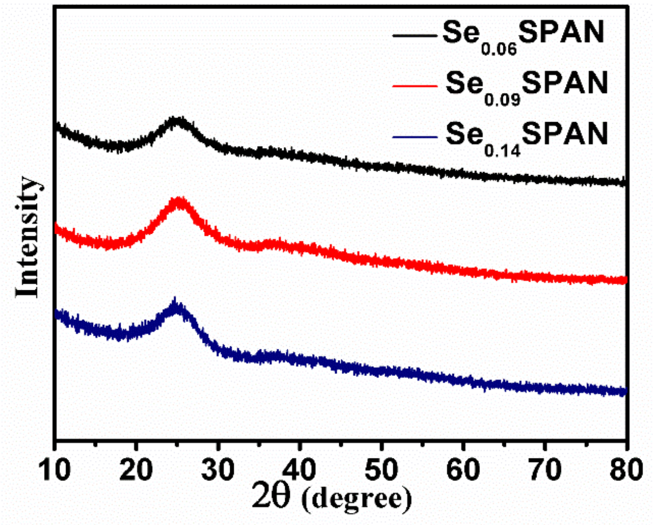

Supplementary Figure 3. **XRD patterns of Se_xSPAN.**

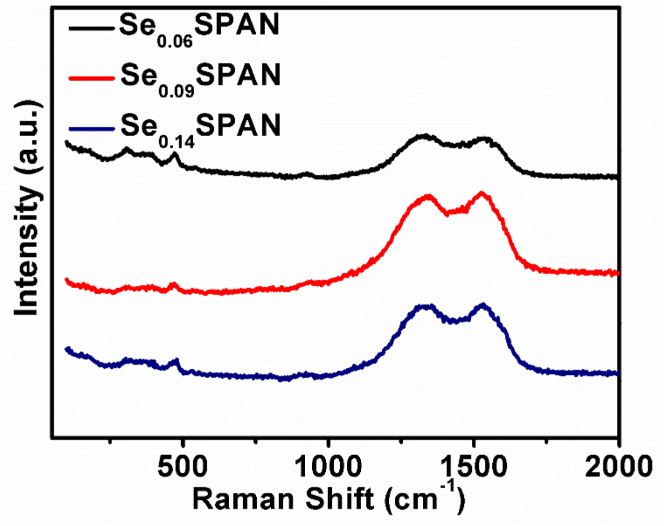

Supplementary Figure 4. **Raman analysis.** Raman Spectra of Se_xSPAN.

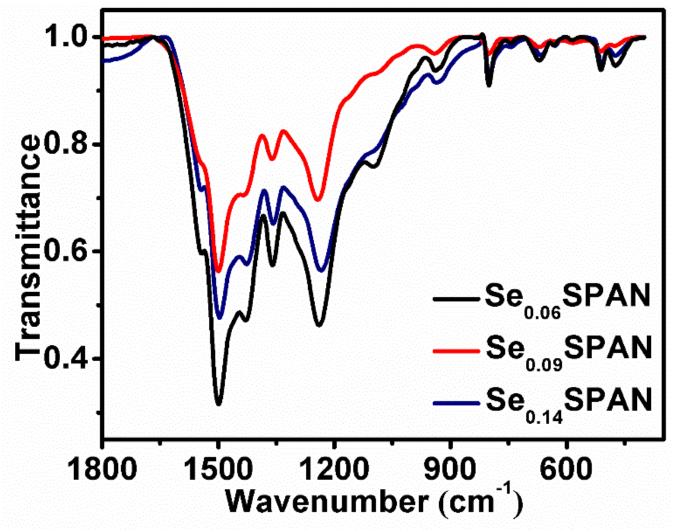

Supplementary Figure 5. **FTIR analysis.** FTIR Spectras of Se_xSPAN composites.

Supplementary Table 4. Raman shifts (cm^{-1}) and assignments for Se_xSPAN.

Se_xSPAN	**Assignments**
**307**	**C-S in plane bending**
**470**	**S-S**
**926**	**Ring (containing S-S bond) Stretch**
**1325**	**D Band**
**1532**	**G Band**

Supplementary Table 5. FTIR wavenumbers (cm^{-1}) and assignments for Se_xSPAN.

Se_xSPAN	**Assignments**
**1543**	**C=C Asymmetric Stretch**
**1498**	**C=C Symmetric Stretch**
**1430**	**C=N Asymmetric Stretch**
**1357**	**C-C Deformation**
**1237**	**C=N Symmetric Stretch**
**940**	**Ring Breath (Side-chain Containing S-S)**
**801**	**Ring Breath (Main-chain Hexahydric-ring)**
**668**	**C-S Stretch**
**511**	**S-S Stretch**
**479**	**S-S**

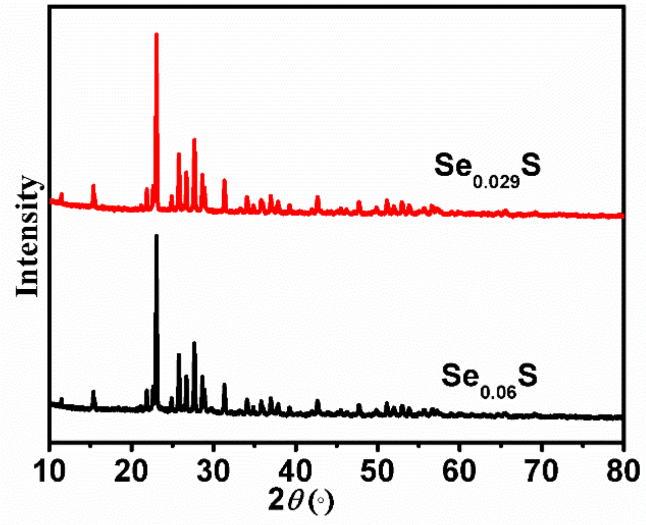

Supplementary Figure 6. **XRD patterns of $Se_{0.029}S$ and $Se_{0.06}S$ composites.**

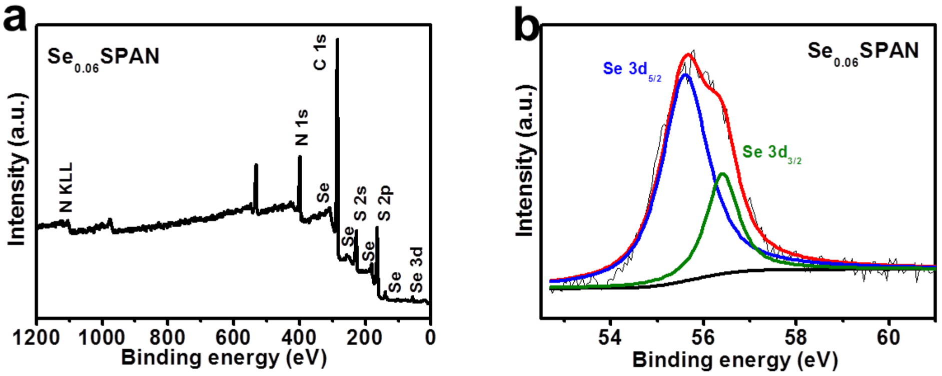

Supplementary Figure 7. **XPS spectra.** (a) Survey XPS spectrum of $Se_{0.06}$SPAN, and (b) Se 3d XPS spectrum.

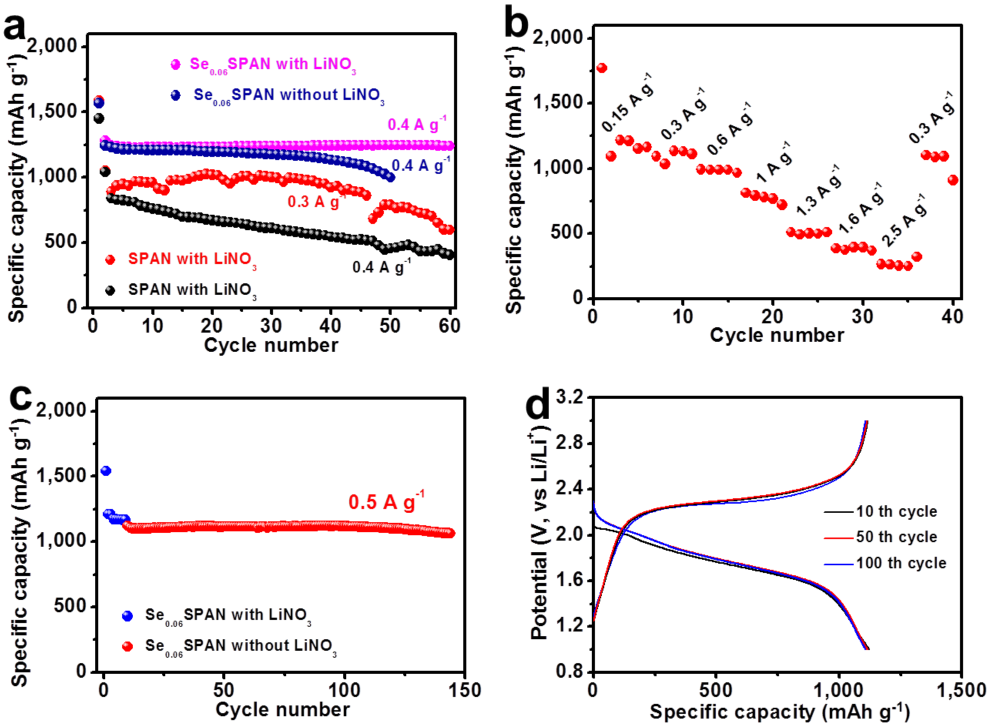

Supplementary Figure 8. **Electrochemical performance of the Li–S batteries.** (a) Cycle performance of the Se_xSPAN cathodes using ether-based electrolyte with and without $LiNO_3$, and SPAN cathode with $LiNO_3$ in ether-based electrolyte. (b) Rate performance of SPAN in the ether-based electrolyte. (c) Cycle performance of the Se_xSPAN cathode using ether-based electrolyte, which is firstly cycled for 10 cycles with $LiNO_3$ and subsequently cycled without $LiNO_3$. (d) The corresponding electrochemical discharge and charge profiles of $Se_{0.06}SPAN$ at various cycles.

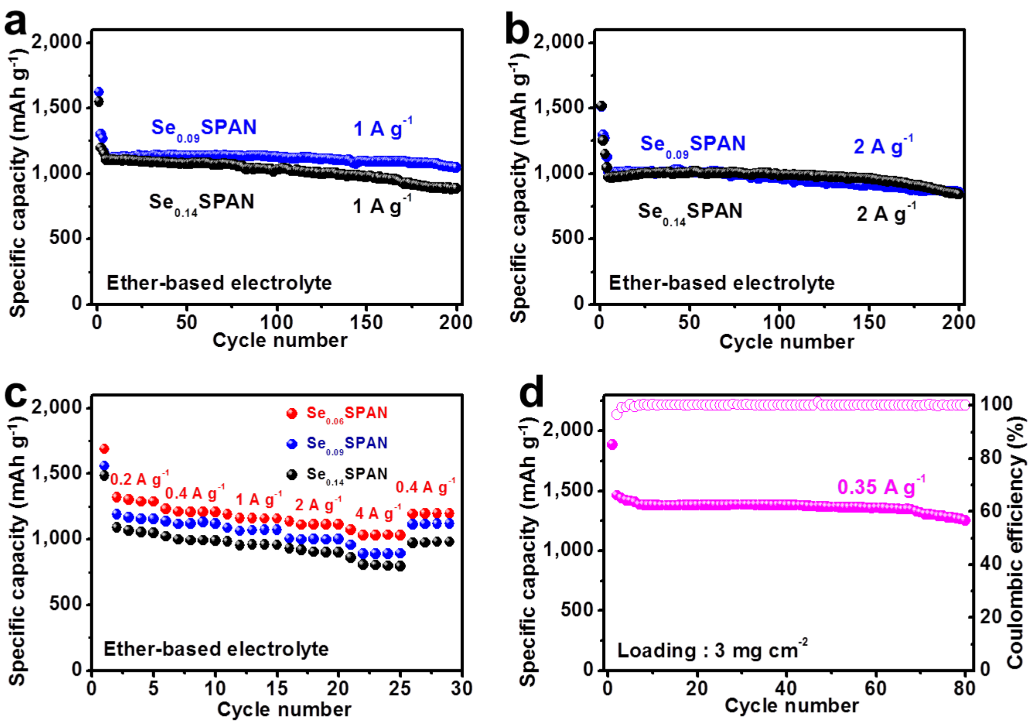

Supplementary Figure 9. **Electrochemical performance of the Li–S batteries.** (a) Cycle performance of $Se_{0.09}SPAN$ and $Se_{0.14}SPAN$ cathodes at 1 A g^{-1}. (b) Cycle performance of $Se_{0.09}SPAN$ and $Se_{0.14}SPAN$ cathodes at 2 A g^{-1}. (c) Rate performance of Se_xSPAN composite cathodes at various current densities. (d) Cycle performance of $Se_{0.06}SPAN$ composite cathode with 3 mg cm^{-2} based on the mass of S and Se in the ether-based electrolyte.

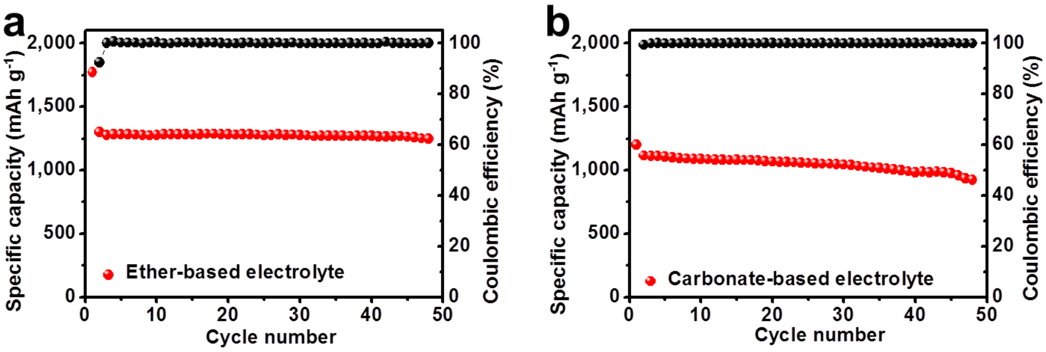

Supplementary Figure 10. **Cycle performance of the Li–S batteries.** $Se_{0.06}$SPAN composite cathodes with 2.3 mg cm^{-2} cycle (a) in the ether-based electrolyte and (b) in the carbonate-based electrolyte after 50 cycles for subsequent tests.

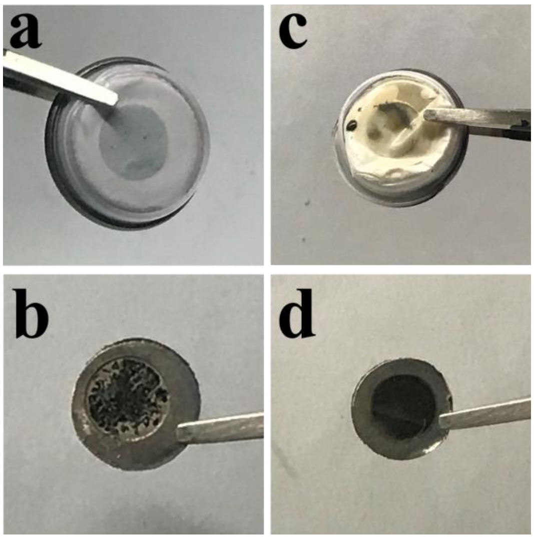

Supplementary Figure 11. **Digital graph of separators and Li metals.** The cycled coin cells were taken in a glovebox after 50 cycles: (a, b) in ether-based electrolyte and (c, d) in carbonate-based electrolyte.

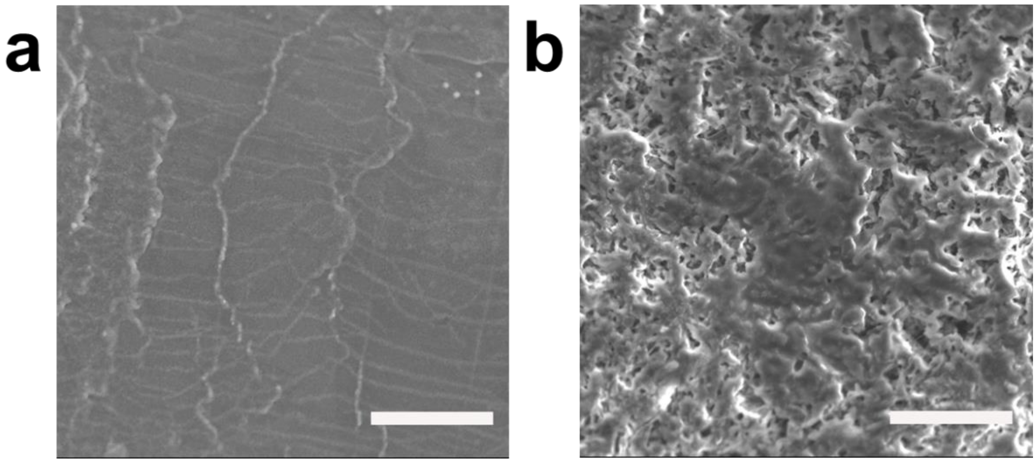

Supplementary Figure 12. Characterization of the cycled Li metal. SEM image of Li metal after 50 cycles: (a) in ether-based electrolyte and (b) in carbonate-based electrolyte. Scale bars, 20 μm (a, b).

Supplementary Table 6 The performance comparison of this work with some similar composite.

Materials[Reference]	Ratio of Active material	Areal loading (mg cm^{-2})	Capacity Retention (mAh g^{-1})	Rate capacity mAh g^{-1}
pPAN-S/GNS[1]	47%	N/A	1200 (0.1 C, 100 cycles)	800 (6 C)
S/PAN/ Mg0.6Ni0.4O[2]	38.5%	1.23	1223 (0.1 C, 100 cycles)	887 (0.5 C); 710 (0.7 C) 445 (1 C)
SPAN/ RGO[3]	44 %	2-2.8	1100 (0.1 C, 200 cycles)	1292 (0.5 C); 1180 (1 C); 828 (2 C)
SPAN[4]	45.6%	~0.38	~1000 (0.4 C, 1000 cycles)	1250 (0.2 C);<br>1125 (0.8 C);<br>1050 (1.6 C)
NiS_2-SPAN[5]	46%	1.15	1533 (0.2 A/g, 100 cycles)	1180 (2 A g^{-1},1.2 C)
MSPAN[6]	45.87%,	2.45	755 (1 C, 200 cycles)	1202 (0.1 C);<br>949 (0.5 C);<br>717 (2 C);<br>350 (5 C)
Fibrous SPAN[7]	43.6%	0.672	N/A	1000 (0.5 C);<br>900 (1 C);<br>750 (2 C);<br>300 (6 C);
$S_{1-x}Se_x/C$[8]	50%,	0.8-1.5	910 (1 A g^{-1}, 500 cycles)	961 (1 A g^{-1}, 0.6 C)<br>926 (2 A g^{-1}, 1.2 C)<br>863 (5 A g^{-1}, 3 C)<br>802 (10 A g^{-1}, 6 C)
$Se_{0.06}$SPAN (This work)	47.25%	1-3	880 (0.4 A g^{-1}, 800 cycles)	1320 (0.2 A g^{-1}, 0.13 C)<br>1140 (2 A g^{-1}, 1.3 C)<br>1070 (4 A g^{-1}, 2.6 C)<br>1000 (6 A g^{-1}, 3.9 C)<br>900 (10 A g^{-1}, 6.5 C)

Supplementary Table 7 Long-term cycling stability of representative cathodes for Li-S batteries.

Materials[Reference]	Initial Capacity (mAh g^{-1})	Capacity Retention (mAh g^{-1})	Decay rate per cycle (%)
CoS_2[9]	1003 (2 C)	321 (2000 cycles)	0.034
MnO_2[10]	934 (2 C)	315 (1700 cycles)	0.039
PEDOT:PSS-graphene[11]	1008 (1 C)	806 (500 cycles)	0.04
Co_9S_8/C[12]	950 (2 C)	560 (1000 cycles)	0.041
CC@Co_3O_4[13]	612 (2 C)	476 (500 cycles)	0.049
Ti_4O_7[14]	850 (2 C)	595 (500 cycles)	0.06
N,S-codoped graphene[15]	878 (0.5 C)	550 (500 cycles)	0.078
ACNF/Co_3S_4/S[16]	953 (1 C)	610 (450 cycles)	0.079
S@Co-N-GC[17]	1150 (1 C)	625 (500 cycles)	0.09
$Se_{0.06}$SPAN (This work)	1230 (0.4 A g^{-1})	880 (800 cycles)	0.029

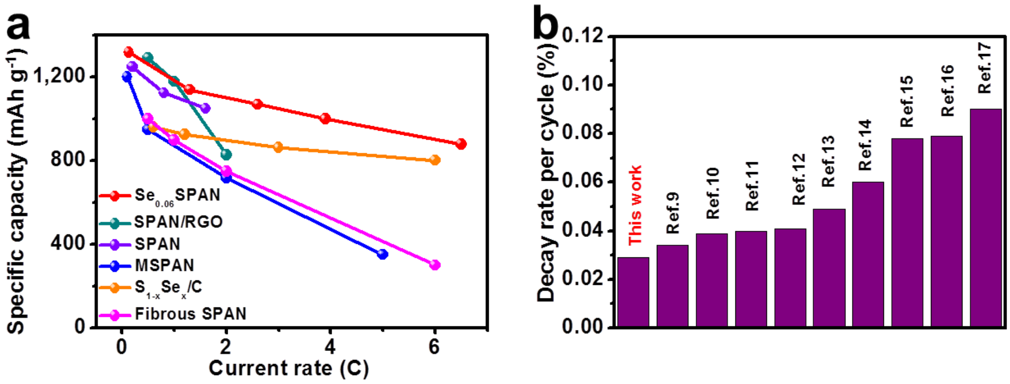

Supplementary Figure 13. **Electrochemical performance comparisons.** (a) Specific capacity and (b) Cyclic life of $Se_{0.06}$SPAN electrodes compared with various SPAN and S based cathode materials.

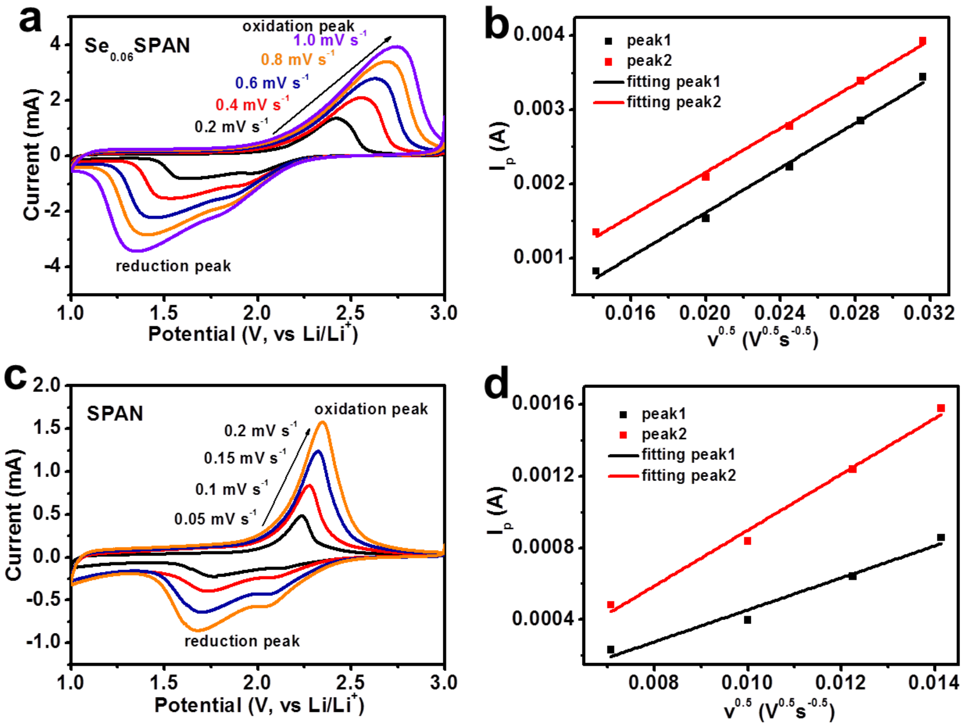

Supplementary Figure 14. CV tests and plots of CV peak current at various scan rates. CV curves and peak currents versus square root of scan rates of (a, b) the $Se_{0.06}SPAN$ and (c, d) SPAN electrodes.

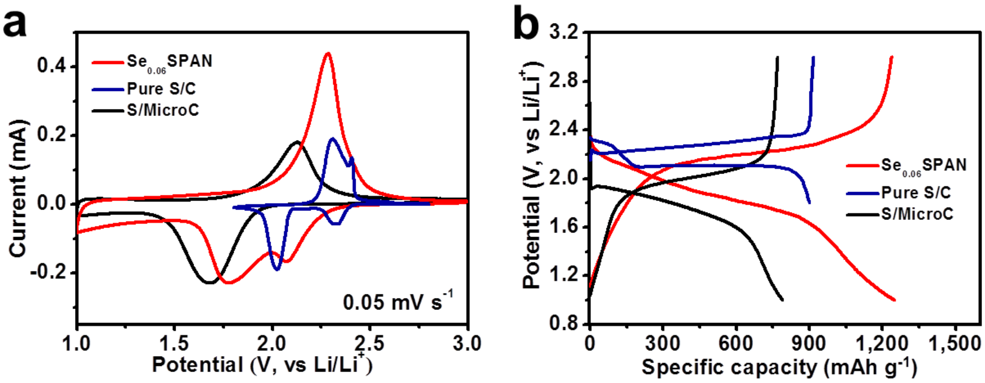

Supplementary Figure 15. **Comparison of CV curves and voltage profiles.** (a) the CV curves and (b) voltage profiles at 0.1 C of Se_xSPAN, Pure S/C and S/microC.

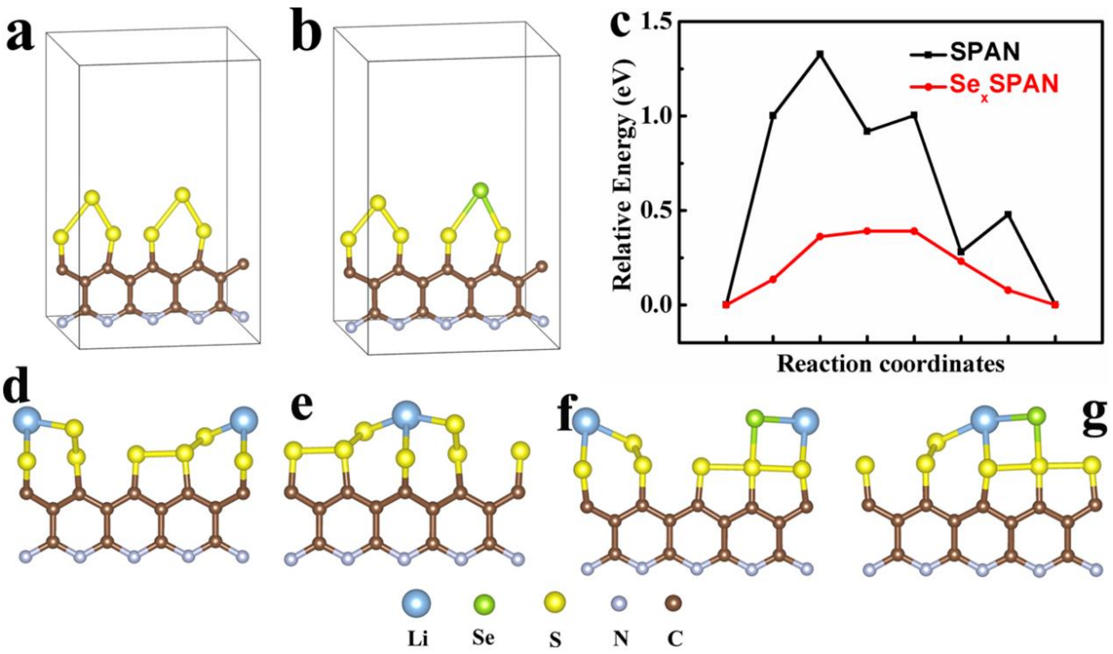

Supplementary Figure 16. **DFT simulation of Li migration behavior.** The model for Li^+ migration energy barriers employing DFT calculation on (a) SPAN and (b) Se_xSPAN. Li, Se, S, N and C atoms are represented by blue, green, yellow, purple and brown spheres, respectively. c) The corresponding energy barriers profiles. Throughout the diffusion path, the Li^+ migration energy barriers on Se_xSPAN is apparently lower than that on SPAN; (d-e) Schematically shows the migration occurs through the following step: Li atom sitting on a S top site passes through a vacant site and to a new S top site. (f-g) Schematically shows the migration occurs through the following step: Li atom sitting on a S top site passes through a vacant site and to a Se top site.

Supplementary References

1. Yin, L., Wang, J., Lin, F., Yang, J. & Nuli, Y. Polyacrylonitrile/graphene composite as a precursor to a sulfur-based cathode material for high-rate rechargeable Li-S batteries. *Energy Environ. Sci.* **5**, 6966-6972 (2012).
2. Zhang, Y., Zhao, Y., Yermukhambetova, A., Bakenov, Z. & Chen, P. Ternary sulfur/polyacrylonitrile/$Mg_{0.6}Ni_{0.4}O$ composite cathodes for high performance lithium/sulfur batteries. *J. Mater. Chem. A* **1**, 295-301 (2013).
3. Li, J. *et al.* A sulfur–polyacrylonitrile/graphene composite cathode for lithium batteries with excellent cyclability. *J. Power Sources* **252**, 107-112 (2014).
4. Wei, S., Ma, L., Hendrickson, K. E., Tu, Z. & Archer, L. A. Metal-sulfur battery cathodes based on PAN–sulfur composites. *J. Am. Chem. Soc.* **137**, 12143-12152 (2015).
5. Liu, Y. *et al.* Polysulfide reduction accelerator-modified sulfurized polyacrylonitrile as a high performance cathode material for lithium-sulfur battery. *J. Mater. Chem. A* 5, 22120-22124 (2017).
6. Liu, Y., Haridas, A. K., Cho, K. K., Lee, Y. & Ahn, J. H. Highly ordered mesoporous sulfurized polyacrylonitrile cathode material for high-rate lithium sulfur batteries. *J. Phys. Chem. C* 121 (47), 26172-26179 (2017).
7. Frey, M. *et al.* Easily accessible, textile fiber-based sulfurized poly(acrylonitrile) as Li/S cathode material: correlating electrochemical performance with morphology and structure. *ACS Energy Letters* **2**, 595-604 (2017).
8. Li, X. *et al.* Amorphous S-rich $S_{1-x}Se_x/C$ ($x\leq0.1$) composites promise better lithium-sulfur batteries in a carbonate-based electrolyte. *Energy Environ. Sci.* **8**, 3181-3186 (2015).
9. Yuan, Z. *et al.* Powering lithium-sulfur battery performance by propelling polysulfide redox at sulfiphilic hosts. *Nano Letters* **16**, 519-527 (2016).
10. Liang, X. & Nazar, L. F. In situ reactive assembly of scalable core-shell sulfur-MnO_2 composite cathodes. *ACS Nano* **10**, 4192-4198 (2016).
11. Xiao, P., Bu, F., Yang, G., Zhang, Y. & Xu, Y. Integration of graphene, nano sulfur, and conducting polymer into compact, flexible lithium-sulfur battery cathodes with ultrahigh volumetric capacity and superior cycling stability for foldable devices. *Adva. Mater.* 1703324 (2017).
12. Chen, T. *et al.* Metallic and polar Co_9S_8 inlaid carbon hollow nanopolyhedra as efficient polysulfide

mediator for lithium-sulfur batteries. *Nano Energy* **38**, 239-248 (2017).

13. Chang, Z. *et al.* Co_3O_4 nanoneedle arrays as a multifunctional "super-reservoir" electrode for long cycle life Li-S batteries. *J. Mater. Chem. A* **5**, 250-257 (2017).
14. Pang, Q., Kundu, D., Cuisinier, M. & Nazar, L. F. Surface-enhanced redox chemistry of polysulphides on a metallic and polar host for lithium-sulphur batteries. *Nat. Commun.* **5**, 4759 (2014).
15. Zhou, G., Paek, E., Hwang, G. S. & Manthiram, A. Long-life Li/polysulphide batteries with high sulphur loading enabled by lightweight three-dimensional nitrogen/sulphur-codoped graphene sponge. *Nat. Commun.* **6**, 7760 (2015).
16. Xu, H. & Manthiram, A. Hollow cobalt sulfide polyhedra-enabled long-life, high areal-capacity lithium-sulfur batteries. *Nano Energy* **33**, 124-129 (2017).
17. Li, Y. J., Fan, J. M., Zheng, M. S. & Dong, Q. F. A novel synergistic composite with multi-functional effects for high-performance Li-S batteries. *Energy Environ. Sci.* **9**, 1998-2004 (2016).
